# Characteristics and overall survival in patients with T1 melanoma: A nationwide matched cohort study

**DOI:** 10.1002/ijc.70287

**Published:** 2025-12-12

**Authors:** Ylva Naeser, Rasmus Mikiver, Karolin Isaksson, Mats Lambe, Gustav J. Ullenhag

**Affiliations:** ^1^ Department of Immunology, Genetics and Pathology Uppsala University Uppsala Sweden; ^2^ Department of Oncology Uppsala University Hospital Uppsala Sweden; ^3^ Department of Biomedical and Clinical Sciences Linköping University Linköping Sweden; ^4^ Department of Biomedical and Clinical Sciences Regional Cancer Center South East Sweden Linköping Sweden; ^5^ Department of Surgery Skåne University Hospital Kristianstad Sweden; ^6^ Department of Clinical Sciences, Surgery Lund University Lund Sweden; ^7^ Department of Medical Epidemiology and Biostatistics Karolinska Institutet Stockholm Sweden

**Keywords:** cohort studies, comorbidity, melanoma, cutaneous malignant, socioeconomic factors, survival

## Abstract

Most cutaneous malignant melanomas (CMMs) are thin (≤1.0 mm, stage T1) with an expected 10‐year melanoma‐specific survival of 93%–97%. The incidence of CMM is higher in groups with high socioeconomic status (SES). We aimed to assess overall survival (OS) and detailed characteristics in individuals with thin CMM as compared to the general population matched on age, sex, and county of residence. Matched cohort study comprising patients diagnosed between 2001 and 2018 with thin CMM (cases) and melanoma‐free comparators from the general population. Patients and comparators were identified in the Malignant Melanoma Data Base Sweden. Multivariable Cox regression analyses were applied to compare the mortality risk for cases and comparators with adjustments for SES and comorbidities. We identified 25,843 cases and 127,383 comparators. Cases had higher SES and less comorbidity. No significant differences in OS were found. However, in the T1a subgroup, comprising 16,941 cases, the 5‐year OS was significantly better than in comparators (*n* = 83,510) (92.5% (95% CI 92.1%–93.0%) versus 91.1% (95% CI 90.8%–91.3%), *p* <.001). The adjusted mortality risk was slightly higher for the whole T1 group (HR 1.05, 95% CI 1.01–1.09), while no difference was found for the T1a subgroup. Deaths attributed to cardiovascular disease, dementia, and chronic obstructive pulmonary disease were less common in CMM patients. Patients diagnosed with thin CMM have an OS similar to or even better than the general population since they are at a lower risk of death from other diseases, likely reflecting socioeconomic and lifestyle factors.

## What's New?

Thin cutaneous malignant melanoma has an excellent ten‐year melanoma‐specific survival of 93%–97%. Moreover, the incidence of melanoma is higher among individuals with high socioeconomic status, which may protect them from other diseases. This nationwide matched cohort study found that patients with thin cutaneous malignant melanoma have a similar survival to the general Swedish population. Higher socioeconomic status and lower risk of dying from dementia, acute myocardial infarction, and chronic obstructive pulmonary disease were characteristics of patients with thin melanoma. These findings may help put into context the health burden of thin cutaneous malignant melanoma on a population level.

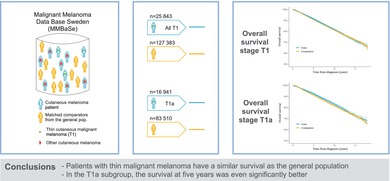

**Abbreviations**
CI: confidence interval
CMM: cutaneous malignant melanoma
HR: hazard ratio
IQR: interquartile range
MMBaSe: Malignant Melanoma data Base Sweden
OS: overall survival
SES: socioeconomic status

## 1 INTRODUCTION

The rapidly increasing incidence of cutaneous malignant melanoma (CMM) is a major health concern worldwide.^1^ Several studies have reported that new CMM cases mainly consist of thin tumours with a Breslow thickness ≤1.0 mm.^2^, ^3^, ^4^ In Sweden, a total of 53,433 new CMMs were registered in the Swedish Melanoma Register during the period 2001–2018; out of these, 29,969 were ≤1.0 mm.

The 10‐year melanoma‐specific survival in patients with thin CMM is in the range of 93%–97%.^5^, ^6^, ^7^, ^8^, ^9^, ^10^ It is even suggested that the overall survival (OS) is close to that of the general population,^7^, ^11^, ^12^, ^13^, ^14^, ^15^ possibly associated with socioeconomic factors. Overdiagnosis of early cutaneous melanomas is also an explanation for both the steeply rising incidence and the excellent outcome.^16^

Lower survival rates have been described for subgroups with ulcerated tumours or Breslow thickness ≥0.8 mm, that is, the rationale for the subdivision of T1 into T1a and T1b in the AJCC 8th edition of melanoma staging.^9^, ^17^, ^18^, ^19^

In addition, 3%–8% of patients diagnosed with a CMM are reported to develop a subsequent CMM.^20^, ^21^, ^22^, ^23^, ^24^, ^25^ Multiple CMMs may enhance the risk of melanoma‐related deaths.^26^, ^27^, ^28^

Men are at higher risk of developing CMM, and women are more often diagnosed with thinner CMMs.^1^, ^29^ Results from several studies also indicate that stage‐specific survival is better in women.^4^, ^7^, ^29^, ^30^, ^31^ Further, the influence of socioeconomic status (SES) is well established, with higher incidence and thinner tumours in groups with high SES.^32^, ^33^, ^34^, ^35^

The aim of this study was to compare OS between individuals diagnosed with a thin CMM (cases) and a matched comparison cohort free of CMM representing the general population. Further, to explore the possible influence of differences in SES and comorbidity burden, including subgroup analyses by sex and for the substage T1a. Finally, to examine and compare the distribution of comorbid conditions in patients with thin CMM and matched comparators.

## 2 MATERIALS AND METHODS

The STROBE reporting guidelines were used.

### 2.1 Data sources and data collection

We used data available in the research database Malignant Melanoma data Base Sweden (MMBaSe), which has previously been described in detail.^36^ The MMBaSe comprises linked data from the Swedish Melanoma Register and several population‐based registers, including the National Patient Register (NPR), the Swedish Cancer Register (SCR), the Cause of Death Register (CDR), and the sociodemographic database (LISA).^36^, ^37^, ^38^, ^39^, ^40^, ^41^

The comparators were randomly selected from the Swedish Population Register (PR) and matched on age, sex, and county of residence at the index date, that is, the date of the thin CMM diagnosis of the case.

We retrieved data on SES and comorbidity at the index date. Data on deaths, including registered underlying causes, and information on malignancies other than CMM for cases and comparators before and after the index date were obtained.

### 2.2 Study population

The cases were defined as individuals ≥18 years with a diagnosis of thin CMM (≤1.0 mm, stage T1) as the first registered diagnosis of CMM between 2001 and 2018. End of follow‐up was 31 December 2018.

All histological subtypes of CMM at all tumour locations were included. The CMM subgroups were defined according to the AJCC8th edition, that is, T1a was defined as a non‐ulcerated CMM <0.8 mm, and T1b as CMM ≥0.8 −≤ 1.0 mm or <0.8 mm with ulceration. Synchronous CMM was an exclusion criterion and was defined as a diagnosis of CMM within 30 days of the index diagnosis recorded in SweMR or the Swedish Cancer Register (SCR). Other reasons for exclusion were: a diagnosis of CMM reported in the SCR, but not in SweMR before the index date, individuals where the personal identifier might have been reused or duplicated, and age below 18 years at the date of diagnosis. If the case was not found in the PR or if no comparators were available, the case was excluded (*N* = 17) (Figure S1).

The comparison cohort comprised up to five comparators free of CMM at the index date. The full multivariable Cox regression analysis for estimation of mortality risk during the study period encompassed cases and matched comparators with all SES and comorbidity variables available (Figure S1).

### 2.3 Socioeconomic status and comorbidity

Three indicators of socioeconomic status were used: highest achieved educational level, income, and marital status. Educational level was categorised into three groups based on the number of years of schooling: low ≤9 years, middle 10–12 years, and high ≥13 years, corresponding to compulsory school, senior high school (secondary, non‐compulsory), and post‐high school (college or university). Household income was defined as disposable income per consumption unit, with the following weights applied by Statistics Sweden: 1.16 for a single adult, 1.92 for two adults, 0.96 for each additional adult >18 years, 0.56 for children 0–3 years, 0.66 for children 4–10 years, and 0.76 for children 0–17 years. Income was then categorised as low (first quartile of the study participants), intermediate (second and third quartiles), or high (fourth quartile). The income quartiles were based on the income of all cases and the mean income of all comparators for each case.

Marital status was divided into four groups: married, unmarried, divorced, or widowed. Comorbidity was assessed according to the Charlson Comorbidity Index (CCI) by use of a CCI algorithm adapted for register‐based research in Sweden, published in 2023.^42^

For cases, all diagnoses registered except CMM until 14 days before the index date were included. For comparators, all diagnoses until the index date were included.

### 2.4 Malignancies

Data on malignancies according to ICD‐7 were obtained for both cases and comparators, both before and after the date of the index diagnosis of the case. All in situ tumors were excluded. The 10 most common diagnoses in cases and comparators, respectively, were assessed. Individuals may have more than one diagnosis.

### 2.5 Cause of death

The underlying cause of death for cases and comparators was obtained according to ICD‐10 and was analysed on a group level for the two major groups: cardiovascular disease (ICD‐10 code I05‐I99) and malignancies (ICD‐10 code C00‐C99), and for the 10 most common separate diagnoses, except for dementia (ICD‐10 codes F03 and G30) and colorectal cancer (ICD‐10 codes C18–C20), which were aggregated.

### 2.6 Statistical analysis

Descriptive statistics were used to characterise cases and comparators at the date of diagnosis of the case (index date). Group comparisons for categorical variables were performed using χ^2^ tests. Continuous variables were summarised as median and interquartile range (IQR) and compared with Mann–Whitney U tests. OS estimates and 95% confidence intervals (CIs) were assessed by the Kaplan–Meier method, with standard errors obtained by Greenwood's formula and CIs based on a log transformation of the survival function. Differences between groups were evaluated by log‐rank tests over 5‐year periods. Adjusted mortality risks were assessed by Cox regression analyses to compare cases and comparators, with results presented as hazard ratios (HRs) with 95% CIs. Cases and comparators with missing variables were excluded. If a case was excluded, the corresponding matched comparators were also excluded from the analyses. The proportional hazards assumption was evaluated using Schoenfeld residuals and was considered reasonably met. The level of significance was set to 0.05, and all *p*‐values were two‐tailed. Bonferroni corrections were applied when multiple testing was performed.

All statistical analyses were performed using R Statistical Software (v4.0.3, R Core Team 2020).

## 3 RESULTS

For the OS analyses, we identified 25,843 patients with a thin CMM as the first diagnosis (cases) and 127,383 comparators. In the subgroup stage T1a, we identified 16,941 patients with a confirmed stage T1a index tumour and 83,510 matched comparators. In the cohort of individuals with thin CMM (cases), 53% (13580) were women. Median age at diagnosis in females was 58 years (interquartile range, IQR 45–70) and 64 years (IQR 52–73) in males. The cases had a significantly higher educational level and disposable income than the comparators. Demographic and socioeconomic characteristics are presented in Table 1.

**TABLE 1:** Demographic, clinical and socioeconomic characteristics of patients with thin (≤1.0 mm) cutaneous malignant melanoma (cases) and their matched comparators.

Parameter	Cases *n* = 25,843 (%)	Comparators *n* = 127,383 (%)	*p*‐value
Sex			.66
Male	12,263 (47)	60,251 (47)	
Female	13,580 (53)	67,132 (53)	
Age, median (IQR)	61 (48–72)	61 (48–71)	.068
Age years			.23
18–39	3193 (12)	15,881 (12)	
40–59	8824 (34)	43,941 (34)	
60–69	6027 (23)	29,803 (23)	
70–79	5135 (20)	25,208 (20)	
80+	2664 (10)	12,550 (9.9)	
Index date (year)			.99
2001–2006	5407 (21)	26,596 (21)	
2007–2012	8097 (31)	39,907 (31)	
2013–2018	12,339 (48)	60,880 (48)	
Marital status			<.001
Married	14,924 (58)	66,108 (52)	
Divorced	3368 (13)	20,913 (16)	
Unmarried	5166 (20)	27,732 (22)	
Widower	2204 (8.5)	12,557 (9.9)	
Missing	157 (0.61)	6 (0.00)	
Educational level			<.001
Low	11,689 (45)	69,265 (54)	
Middle	7950 (31)	33,818 (27)	
High	5885 (23)	22,586 (18)	
Missing	319 (1.2)	1714 (1.4)	
Disposable income (family)			<.001
Low	5237 (20)	37,795 (30)	
Intermediate	13,107 (51)	62,083 (49)	
High	7270 (28)	27,505 (22)	
Missing	229 (0.89)	0 (0.0)	
Charlson Comorbidity Index (CCI)			<.001
0	19,558 (76)	95,594 (75)	
1	4350 (17)	21,175 (17)	
+ 2	1935 (7.5)	10,614 (8.3)	
Separate CCI diagnoses			
Congestive heart failure	829 (3.2)	4403 (3.5)	.047
Peripheral vascular disease	430 (1.7)	2566 (2.0)	<.001
Cerebrovascular disease	1384 (5.4)	7138 (5.6)	.12
Chronic obstructive pulmonary disease	369 (1.4)	2745 (2.2)	<.001
Chronic other pulmonary disease	708 (2.7)	4195 (3.3)	<.001
Rheumatic disease	668 (2.6)	3057 (2.4)	.082
Dementia	142 (0.55)	1441 (1.1)	<.001
Hemiplegia	132 (0.51)	759 (0.60)	.11
Diabetes without chronic complication	24 (0.090)	156 (0.12)	.24
Diabetes with chronic complication	467 (1.8)	2865 (2.3)	<.001
Renal disease	289 (1.1)	1521 (1.2)	.32
Mild liver disease	57 (0.22)	788 (0.62)	<.001
Liver special	14 (0.050)	90 (0.070)	.43
Severe liver disease	21 (0.080)	169 (0.13)	.041
Peptic ulcer disease	307 (1.2)	2038 (1.6)	<.001
Malignancy^a^	2133 (8.3)	8245 (6.5)	<.001
Metastatic solid cancer	287 (1.1)	1062 (0.83)	<.001
AIDS	5 (0.020)	73 (0.060)	.021

*Note*: Number of participants (%) unless otherwise specified. IQR, interquartile range; Education: categorised according to years of schooling: low ≤9 years, middle 10–12 years and high ≥13 years. Income: based on total income for the entire study population (disposable income per consumption unit); Low: first quartile Q1, Intermediate: Q2 + Q3, High: Q4. CCI at baseline: For cases, all diagnoses registered in the National Patient Register until 14 days before the diagnosis of the index diagnosis of cutaneous malignant melanoma were included. For comparators, all diagnoses until the index date were included.

^a^ In cases the index CMM diagnosis was excluded. Level of significance was set to *p* <.05 for sociodemographic parameters and to *p* <.003 for separate CCI diagnoses (according to Bonferroni correction 0.05/18).

The full multivariable Cox regression analysis for estimation of mortality risk during the study period encompassed 25,424 cases and 124,014 matched comparators with all SES and comorbidity variables available. For the T1a subgroup analysis, the corresponding numbers were 16,674 cases and 81,334 matched comparators.

A detailed flowchart is presented in Figure S1.

### 3.1 Characteristics of the index tumours

Median tumour Breslow thickness was 0.6 mm (IQR 0.4–0.8). Of all index tumours, 66% (16941) were classified as stage T1a and 28% (7325) as stage T1b. Ulceration status was not registered for 1577 tumours (6.1%).

### 3.2 Comorbidity

The overall comorbidity burden at the index date was lower in cases, with significantly lower prevalence of seven of the 18 diagnosis groups included in the CCI. Malignancies were the most common concomitant conditions in both cases (8.3%) and in comparators (6.5%). Metastatic solid tumors were significantly more common in cases (Table 1). Of note, non‐melanoma skin cancer was not included in the CCI algorithm.

### 3.3 Malignancies before index date

The proportion of individuals in the thin CMM patient group diagnosed with any malignancy, including non‐melanoma skin cancer, was 12% (3170) compared to 8.8% (11,171) in the comparison cohort. The two most common malignancies in both cases and comparators were prostate cancer (2.9% (759) versus 2.3% (2914)) and breast cancer (2.5% (634) versus 1.8% (2253)). The 10 most frequent sites were similar in cases and comparators, and out of these, the prevalence in cases was significantly higher in eight (Table 2).

**TABLE 2:** Distribution of malignancies in patients with thin (≤1.0 mm) cutaneous malignant melanoma (cases) and their matched comparators before and after the index diagnosis of the cases.

	Cases *n* (%) before index date	Comparators *n* (%) before index date	*p*‐value	Cases *n* (%) after index date	Comparators *n* (%) after index date	*p*‐value
All malignancies (140–209)	3170 (12)	11,171 (8.8)		3527 (14)	1053 (8.1)	
Cutaneous malignant melanoma (190)	NA	NA	NA	995 (3.9)	635 (0.50)	<.001
Prostate cancer (177)	759 (2.9)	2914 (2.3)	<0.001	548 (2.1)	2047 (1.6)	<.001
Breast cancer (170)	634 (2.5)	2253 (1.8)	<0.001	299 (1.2)	1234 (0.97)	.0060
Non‐melanoma skin cancer (191)	442 (1.7)	827 (0.65)	<0.001	689 (2.7)	1025 (0.80)	<.001
Colorectal cancer (153–154)	309 (1.2)	1279 (1.0)	0.0060	271 (1.0)	1248 (0.98)	.33
Urinary tract cancer (181)	153 (0.59)	713 (0.56)	0.56	119 (0.46)	601 (0.47)	.85
Uterine cancer (172)	132 (0.51)	467 (0.37)	0.001	54 (0.21)	246 (0.19)	.65
Non‐Hodgkin lymphoma (200)	129 (0.50)	343 (0.27)	<0.001	92 (0.36)	343 (0.27)	.020
Endocrine gland cancer (195)	112 (0.43)	366 (0.29)	<0.001	53 (0.21)	170 (0.13)	.0080
Cancer in the nervous system (193)	106 (0.41)	350 (0.27)	<0.001	66 (0.26)	256 (0.20)	.095
Kidney cancer (180)	81 (0.31)	272 (0.21)	0.003	68 (0.26)	248 (0.19)	.033
Lung cancer (162)	63 (0.24)	228 (0.18)	0.035	172 (0.67)	847 (0.66)	1.0
Pancreatic cancer (157)	7 (0.027)	27 (0.021)	0.73	48 (0.19)	277 (0.22)	.35

*Note*: All malignancies include ICD‐7 codes 140‐209. All in situ tumours were excluded. Total number of individuals and percentage of cases and comparators respectively who were diagnosed with each malignancy. An individual may have more than one diagnosis. The 10 most common cancers before and after the index date in cases and their comparators were included in the table. Level of significance was set to *p* <.004 (according to Bonferroni correction 0.05/13).

### 3.4 Malignancies after index date

The proportion of individuals subsequently diagnosed with any malignancy was 14% (3527) in cases with thin CMM and 8.1% (10,353) in comparators.

Distribution of malignancies by site showed a somewhat different pattern than prior to the index date (Table 2).

However, none of the 18 most common diagnoses was significantly more frequent in comparators than in cases, neither before nor after the index date.

### 3.5 Overall survival

Median duration of follow‐up was 66 months for the cases (IQR 30–117) and 65 months for the comparators (IQR 30–117). The proportion of deceased individuals was 13% (3367) in the thin CMM patient group and 13% (16,874) in the matched cohort during the study period. In the subgroup stage T1a, the corresponding proportions were 11% (1893) in cases and 12% (10,056) in comparators.

### 3.6 Survival in thin melanoma patients and matched comparators

**FIGURE 1:**
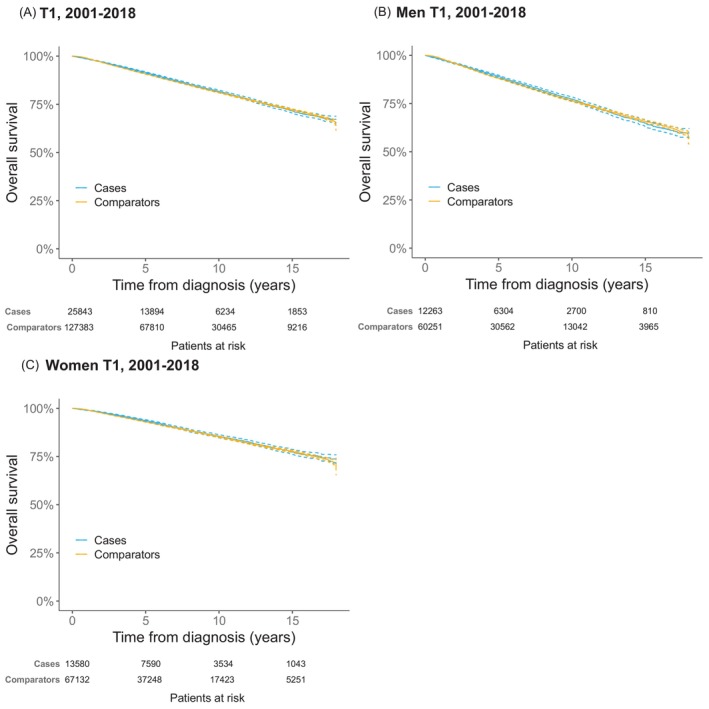
Overall survival in patients with thin (≤1.0 mm) cutaneous malignant melanoma as first diagnosis reported in the Malignant Melanoma Data Base Sweden (MMBaSe) between 2001 and 2018 (blue) and their matched comparators (yellow). Dashed lines represent 95% CI. (A) Overall survival in all patients and matched comparators. (B) Overall survival in male patients and matched comparators. (C) Overall survival in female patients and matched comparators.

OS in patients with thin CMM was similar to that of the matched comparison group. In the subgroup analyses by sex, a generally better OS was noted in women, but without significant differences between cases and their matched comparators (Figure 1). Estimates for the different diagnosis periods are shown in Figures S2–S4.

### 3.7 Survival analyses in the stage T1a subgroup

**FIGURE 2:**
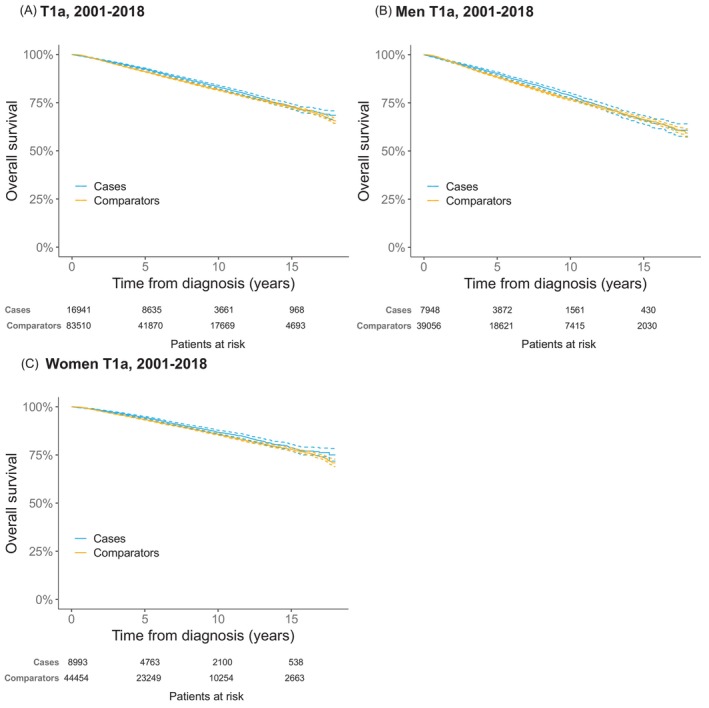
Overall survival in patients with stage T1a cutaneous malignant melanoma as first diagnosis reported in the Malignant Melanoma Data Base Sweden (MMBaSe) between 2001 and 2018 (blue) and their matched comparators (yellow). Dashed lines represent 95% CI. (A) Overall survival in all patients and matched comparators. (B) Overall survival in male patients and matched comparators. (C) Overall survival in female patients and matched comparators.

In individuals with stage T1a disease, there was a small but significant difference in OS between T1a patients and comparators at 5 years; 92.5% (95% CI 92.1%–93.0%) of cases were alive versus 91.1% (95% CI 90.8%–91.3%) of the comparators (*p* <.001). At 10 and 15 years, Kaplan–Meier estimates showed no clear differences in OS, as the confidence intervals overlapped (Figure 2). Estimates for the different diagnostic periods are available in Figures S5–S7.

In separate analyses by sex, a small but significant difference was found in OS in male cases compared to their comparators at 5 years, 90.3% (95% CI 89.5%–91.0%) and 88.4% (95% CI 88.0%–88.7%) were alive respectively (*p* <.001). In women, the difference was even smaller with 94.5% (95% CI 93.9%–95.0%) of the cases alive vs. 93.4% (95% CI 93.1%–93.7%) of the comparators (*p* = .001). At 10 and 15 years, no clear differences in OS were observed between male or female cases and their comparators (Figure 2).

### 3.8 Mortality

#### 3.8.1 Univariable and multivariable analysis

In univariable Cox proportional hazard regression analysis, the overall mortality risk was similar in the thin CMM patient group compared to the matched cohort. In the T1a patient subgroup, the overall mortality risk was significantly lower in cases (HR 0.917, 95% CI 0.873–0.963) compared to the matched cohort. Results were similar in men and women.

In a model adjusted for SES and comorbidity, a slightly higher risk of death was noted in the thin CMM patient group compared to the matched cohort (HR 1.05, 95% CI 1.01–1.09). A similar result was observed in men (HR 1.07, 95% CI 1.01–1.12). In women, the corresponding estimate was HR 1.03 (95% CI 0.975–1.10) (Table S1).

In the T1a patient subgroup, no significant differences in the risk of death were noted between cases and the matched cohort in men and women combined (HR 0.988, 95% CI 0.939–1.04) or in men (HR 1.01, 95% CI 0.947–1.08) or in females (HR 0.963, 95% CI 0.890–1.04) alone. (Table S2).

### 3.9 Cause of death

In both individuals with thin CMM and their comparators, the two major underlying causes of death on an aggregated level were cardiovascular disease and malignancies, although the order of these causes differed (Table 3).

**TABLE 3:** Underlying cause of death in patients with thin (≤1.0 mm) cutaneous malignant melanoma (cases) and their matched comparators: Grouped and most common causes of death.

	Cases *n* (%)	Comparators *n* (%)	*p*‐value
All‐cause mortality	3367 (100)	16,874 (100)	
Cause of death (ICD‐10 code)			
Malignancy (C00‐C99)	1388 (41)	4378 (26)	<.001
Cutaneous malignant melanoma (C43)	481 (14)	70 (0.41)	<.001
Lung cancer (C34)	134 (4.0)	732 (4.3)	.37
Prostate cancer (C61)	113 (3.4)	580 (3.4)	.85
Colorectal cancer (C18‐C20)	102 (3.0)	498 (3.0)	.85
Pancreatic cancer (C25)	67 (2.0)	344 (2.0)	.91
Cardiovascular disease (I05‐I99)	1020 (30)	6287 (37)	<.001
Chronic ischemic heart disease (I25)	210 (6.2)	1251 (7.4)	.018
Acute myocardial infarction (I21)	167 (5.0)	1250 (7.4)	<.001
Heart failure (I50)	137 (4.1)	643 (3.8)	.51
Atrial fibrillation and flutter (I48)	86 (2.6)	435 (2.6)	.98
Dementia (F03, G30)	172 (5.1)	1153 (6.8)	<.001
Chronic obstructive pulmonary disease (J44)	75 (2.2)	539 (3.2)	.003

*Note*: Cause of death: Underlying cause of death registered in the Cause of Death Register according to International Classification of Diseases, Tenth Revision (ICD‐10). Number of individuals and distribution of cause of death in percent (%) among deceased individuals in each category. The 10 most common causes of death in cases and comparators are included in the table. Level of significance was set to *p* <.004 (according to Bonferroni correction 0.05/13).

In cases, CMM was the overall leading cause of death by 14%. A total of 481 individuals, corresponding to 1.9% of all CMM patients, died of CMM. Of the diseased cases, CMM was registered as the cause of death in 9.8% (185) of individuals with T1a, 22% (248) of the group with T1b, and 15% (48) in the group T1 unclassified.

In comparators, the most common causes of death were chronic ischemic heart disease (7.4%) and acute myocardial infarction (7.4%).

Of the 10 most common causes of death in cases and comparators, the cases significantly more often died of CMM, while death due to dementia, including Alzheimer's disease, acute myocardial infarction, and chronic obstructive pulmonary disease, was significantly more common in the comparison cohort (Table 3).

## 4 DISCUSSION

We used information in the Swedish research database MMBaSe to in detail compare the OS in patients diagnosed with CMM, ≤1.0 mm, with matched comparators representing the general population. We found that individuals diagnosed with a thin CMM had a similar survival as the matched comparison cohort at 5, 10, and 15 years of follow‐up. Previous studies have shown that melanoma incidence is higher in high‐ compared to low SES groups.^34^, ^35^, ^43^ In our study, individuals with thin CMM had significantly higher SES than their comparators. Therefore, we also investigated whether the risk of death was affected by socio‐economic factors and comorbidity burden. In a model adjusted for these factors, a slightly higher risk of death was observed in patients with thin CMM.

Some significant differences were observed in the distribution of registered underlying causes of death. Although less than 2% of the thin CMM patients had CMM as the recorded cause of death, this corresponded to a 35‐fold higher risk of melanoma‐related death than in comparators. On the other hand, conditions that were significantly less common as causes of death in cases were dementia, acute myocardial infarction, and chronic obstructive pulmonary disease.

Interestingly, a history of malignant disease was more common in patients with thin CMM, and malignancies were also more common in CMM patients compared to comparators after the index date. A subsequent CMM was diagnosed in 3.9% of individuals with thin CMM, corresponding to a nearly eight times higher risk than in the comparators. Also, non‐melanoma skin cancer was clearly more common among cases both before and after the index date.

When excluding melanoma and non‐melanoma skin cancer, the three most common cancers in cases and comparators were prostate cancer, colorectal cancer, and breast cancer. Of these, prostate cancer and breast cancer were significantly more common in cases before the index date; prostate cancer was also more common after the index date, confirming findings in earlier studies.^44^, ^45^ Although we found that other malignancies were more common in patients with thin CMM, non‐melanoma cancer‐related deaths were not more common than in comparators. This finding is likely to reflect a tendency to seek early professional advice for signs or symptoms of disease, leading to early detection. Also, in line with findings for CMM, the prognosis of prostate cancer and breast cancer generally is better in high SES groups.^46^, ^47^

In a subgroup analysis, we found that patients with CMM stage T1a experienced a slightly better survival than their comparators at 5 years. However, in a model estimating the mortality risk adjusted for SES and comorbidity (CCI), no significant difference remained.

We have previously reported a significantly better OS in patients with melanoma in situ compared to the general population, a finding suggested to reflect differences in SES, lifestyle factors, and health‐seeking behaviours. In that study, CMM was noted as the cause of death in 3.5% of deceased cases.^36^ Although our two studies are not directly comparable, they provide evidence that being diagnosed with an early‐stage melanoma is associated with characteristics associated with a generally lower risk of death. A CMM ≤1.0 mm appears to constitute the break‐even point when the increased risk of dying from CMM equals the reduced risk of dying from other diseases.

Strengths of the present study included the population‐based setting with individual‐level data retrieved from nationwide registers of high quality and completeness. However, there are several limitations. Previous studies have shown that age has an impact on prognosis. Age‐stratified analyses were not performed in this study due to the design with age‐matched comparators.

No data were available on factors that may influence both the risk of CMM and the risk and prognosis of other diseases. These include life‐style factors such as smoking, sun exposure, dietary habits, health care seeking behaviour, detailed medical history, and ethnicity. In the absence of primary care data, the comorbidity burden in both cases and comparators was most likely underestimated. Furthermore, since half of the patients were diagnosed between 2013 and 2018, the median follow‐up time was somewhat short, 5.5 years, making survival estimates beyond 10 years uncertain. A censoring rate of 85% should be noted. However, as shown in the supplement figures, similar OS results were noted in cases and comparators across all calendar periods of diagnosis. Hence, we do not believe that the relatively short follow‐up time and the high censoring rate have biased the survival results.

The OS in individuals diagnosed with a thin CMM is similar and for the stage T1a subgroup even significantly better at 5 years compared to the general population. Our findings should be considered when informing these patients and when discussing the health burden of thin CMMs on a population level. In view of having a ‘normal’ expected lifetime, the need for intense follow‐up programmes for this patient group is questionable. Furthermore, our results may counteract negative social implications of the malignant diagnosis, such as discrimination when taking out insurances.

## AUTHOR CONTRIBUTIONS

**Ylva Naeser:** Writing – original draft; investigation; project administration. **Rasmus Mikiver:** Writing – review and editing; formal analysis; investigation. **Karolin Isaksson:** Writing – review and editing; investigation. **Mats Lambe:** Methodology; writing – review and editing; investigation. **Gustav J. Ullenhag:** Funding acquisition; writing – original draft; supervision; conceptualization; investigation.

## FUNDING INFORMATION

This study was supported by grants from Stiftelsen Onkologiska Klinikens i Uppsala Forskningsfond, Region Skåne (ALF) and Uppsala University Hospital (ALF).

## CONFLICT OF INTEREST STATEMENT

None of the authors have any competing interests to declare.

## ETHICS STATEMENT

Ethical approval was granted by the Regional Ethics Board in Uppsala (# 2018/405) for the construction of MMBaSe and associated study projects.

## Supporting information


**Supplementary Table 1.** Multivariable Cox proportional regression of overall.
**Supplementary Table 2**. Multivariable Cox proportional regression of overall.
**Supplementary Figure 1**. Final study population for thin cutaneous malignant.
**Supplementary Figure 2**. Overall survival: Patients with thin cutaneous malignant.
**Supplementary Figure 3**. Overall survival: Patients with thin cutaneous malignant.
**Supplementary Figure 4**. Overall survival: Patients with thin cutaneous malignant.
**Supplementary Figure 5**. Overall survival: Patients with stage T1a cutaneous.
**Supplementary Figure 6**. Overall survival: Patients with stage T1a cutaneous.
**Supplementary Figure 7**. Overall survival: Patients with stage T1a cutaneous.

## Data Availability

De‐identified data that support the findings of this study are available from the corresponding author upon reasonable request.
