# Supplementary material for: Characteristics and overall survival in patients with T1 melanoma: A nationwide matched cohort study
Source: Int J Cancer. 2025 Dec 12;158(10):2581–91. doi: 10.1002/ijc.70287 (PMC12996739; doi:10.1002/ijc.70287)
Supplement: Supplementary file 1 — Supplementary Table 1. Multivariable Cox proportional regression of overall. Supplementary Table 2. Multivariable Cox proportional regression of overall. Supplementary Figure 1. Final study population for thin cutaneous malignant. Supplementary Figure 2. Overall survival: Patients with thin cutaneous malignant. Supplementary Figure 3. Overall survival: Patients with thin cutaneous malignant. Supplementary Figure 4. Overall survival: Patients with thin cutaneous malignant. Supplementary Figure 5. Overall survival: Patients with stage T1a cutaneous. Supplementary Figure 6. Overall survival: Patients with stage T1a cutaneous. Supplementary Figure 7. Overall survival: Patients with stage T1a cutaneous. [file IJC-158-2581-s001.pdf]

Characteristics and overall survival in patients with T1 melanoma. A nationwide matched cohort study

Ylva Naeser, Rasmus Mikiver, Karolin Isaksson, Mats Lambe and Gustav J. Ullenhag

Table of contents

Supplementary Table 1. Multivariable Cox proportional regression of overall mortality risk in patients with thin cutaneous malignant melanoma (cases) and their matched comparators.

Supplementary Table 2. Multivariable Cox proportional regression of overall mortality risk in patients with stage T1a cutaneous malignant melanoma (cases) and their matched comparators.

Supplementary Figure 1. Final study population for thin cutaneous malignant melanoma including the subgroup stage T1a for analysis of survival and Cox regression analysis and reasons for exclusion for cases and their matched comparators (flow chart).

Supplementary Figure 2. Overall survival: Patients with thin cutaneous malignant melanoma as first diagnosis, diagnosed between 2001 and 2006, and their matched comparators.

Supplementary Figure 3. Overall survival: Patients with thin cutaneous malignant melanoma as first diagnosis, diagnosed between 2007 and 2012, and their matched comparators.

Supplementary Figure 4. Overall survival: Patients with thin cutaneous malignant melanoma as first diagnosis, diagnosed between 2013 and 2018, and their matched comparators.

Supplementary Figure 5. Overall survival: Patients with stage T1a cutaneous malignant melanoma as first diagnosis, diagnosed between 2001 and 2006, and their matched comparators.

Supplementary Figure 6. Overall survival: Patients with stage T1a cutaneous malignant melanoma as first diagnosis, diagnosed between 2007 and 2012, and their matched comparators.

Supplementary Figure 7. Overall survival: Patients with stage T1a cutaneous malignant melanoma as first diagnosis, diagnosed between 2013 and 2018, and their matched comparators.

Supplementary Table 1. Multivariable Cox proportional regression of overall mortality risk in patients with thin (≤ 1.0 mm) cutaneous malignant melanoma (cases) and their matched comparators.

	**All study participants Overall mortality**		**Men Overall mortality**		**Women Overall mortality**	
**Variable**	**Hazard ratio**	**95% CI**	**Hazard ratio**	**95% CI**	**Hazard ratio**	**95% CI**
**Comparators**	1	Reference	1	Reference	1	Reference
**Cases**	1.05	1.01-1.09	1.07	1.01-1.12	1.03	0.975-1.10
**Education**						
Low	1	Reference	1	Reference	1	Reference
Middle	0.651	0.626-0.678	0.782	0.746-0.819	0.450	0.418-0.484
High	0.566	0.536-0.597	0.677	0.632-0.726	0.496	0.455-0.541
**Charlson Comorbidity index**						
0	1	Reference	1	Reference	1	Reference
1	3.09	2.99-3.20	2.96	2.83-3.10	2.93	2.78-3.09
2	6.95	6.69-7.21	6.18	5.88-6.48	6.25	5.87-6.64
**Marital status**						
Married	1	Reference	1	Reference	1	Reference
Divorced	0.979	0.939-1.02	1.03	0.972-1.08	1.13	1.05-1.20
Unmarried	0.657	0.627-0.689	0.664	0.627-0.702	0.699	0.645-0.758
Widower	2.35	2.26-2.44	2.33	2.20-2.46	3.26	3.08-3.44
**Disposable income**						
Low	1	Reference	1	Reference	1	Reference
Intermediate	0.629	0.609-0.648	0.572	0.549-0.596	0.621	0.591-0.652
High	0.450	0.425-0.476	0.383	0.355-0.412	0.456	0.417-0.500

Hazard ratios and 95% confidence intervals (CI) for overall mortality during the study period. All variables are mutually adjusted. Income: based on total income for the entire study population (disposable income per consumption unit). Low: first quartile Q1, Intermediate Q2+Q3, High Q4.

Supplementary Table 2. Multivariable Cox proportional regression of overall mortality risk in patients with stage T1a cutaneous malignant melanoma (cases) and their matched comparators.

	All study participants Overall mortality		Men Overall mortality		Women Overall mortality	
**Variable**	**Hazard ratio**	**95% CI**	**Hazard ratio**	**95% CI**	**Hazard ratio**	**95% CI**
**Comparators**	1	Reference	1	Reference	1	Reference
**Cases**	0.988	0.939-1.04	1.01	0.947-1.08	0.963	0.890-1.04
**Education**						
Low	1	Reference	1	Reference	1	Reference
Middle	0.650	0.618-0.684	0.785	0.738-0.834	0.439	0.399-0.483
High	0.546	0.509-0.586	0.666	0.608-0.728	0.461	0.412-0.516
**Charlson Comorbidity index**						
0	1	Reference	1	Reference	1	Reference
1	3.09	2.96-3.24	2.92	2.75-3.10	2.97	2.77-3.18
2	7.00	6.67-7.34	6.26	5.88-6.66	6.16	5.69-6.66
**Marital status**						
Married	1	Reference	1	Reference	1	Reference
Divorced	0.971	0.920-1.02	1.02	0.956-1.10	1.12	1.02-1.22
Unmarried	0.648	0.610-0.688	0.668	0.620-0.719	0.675	0.608-0.750
Widower	2.33	2.22-2.44	2.36	2.20-2.54	3.21	2.99-3.45
**Disposable income**						
Low	1	Reference	1	Reference	1	Reference
Intermediate	0.623	0.598-0.648	0.558	0.530-0.588	0.636	0.597-0.678
High	0.435	0.404-0.468	0.358	0.324-0.395	0.480	0.427-0.541

Hazard ratios and 95% confidence intervals (CI) for overall mortality during the study period. All variables are mutually adjusted. Income: based on total income for the entire study population (disposable income per consumption unit). Low: first quartile Q1, Intermediate Q2+Q3, High Q4.

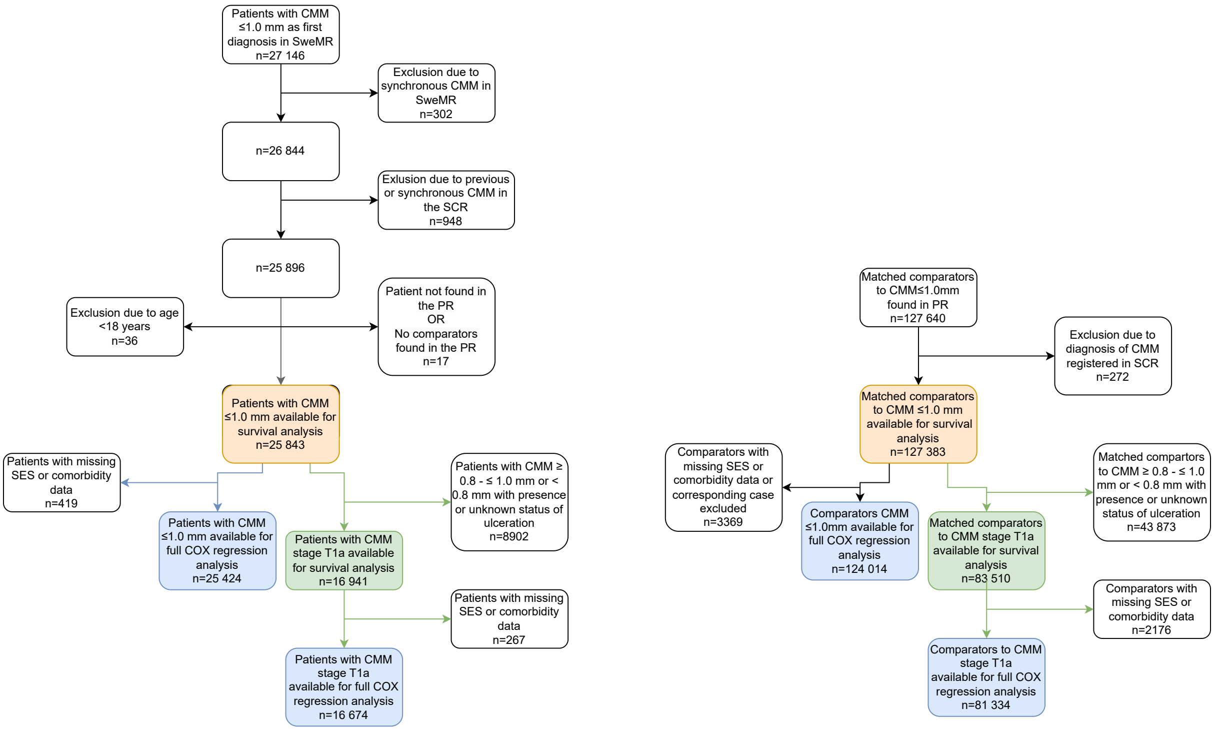

Supplementary Figure 1. All individuals with a diagnosis of thin (≤ 1.0 mm) cutaneous malignant melanoma as first diagnosis reported in Malignant Melanoma Data Base Sweden (MMBaSe) between 2001 and 2018 were included in the present study. Final study population for thin cutaneous malignant melanoma including the subgroup stage T1a for analysis of survival and Cox regression analysis and reasons for exclusion for cases and their matched comparators. Abbreviations used: The Swedish Melanoma Register (SweMR), Cutaneous Malignant Melanoma (CMM), the Swedish Cancer Register (SCR), Socioeconomic Status (SES) and the Population Register (PR).

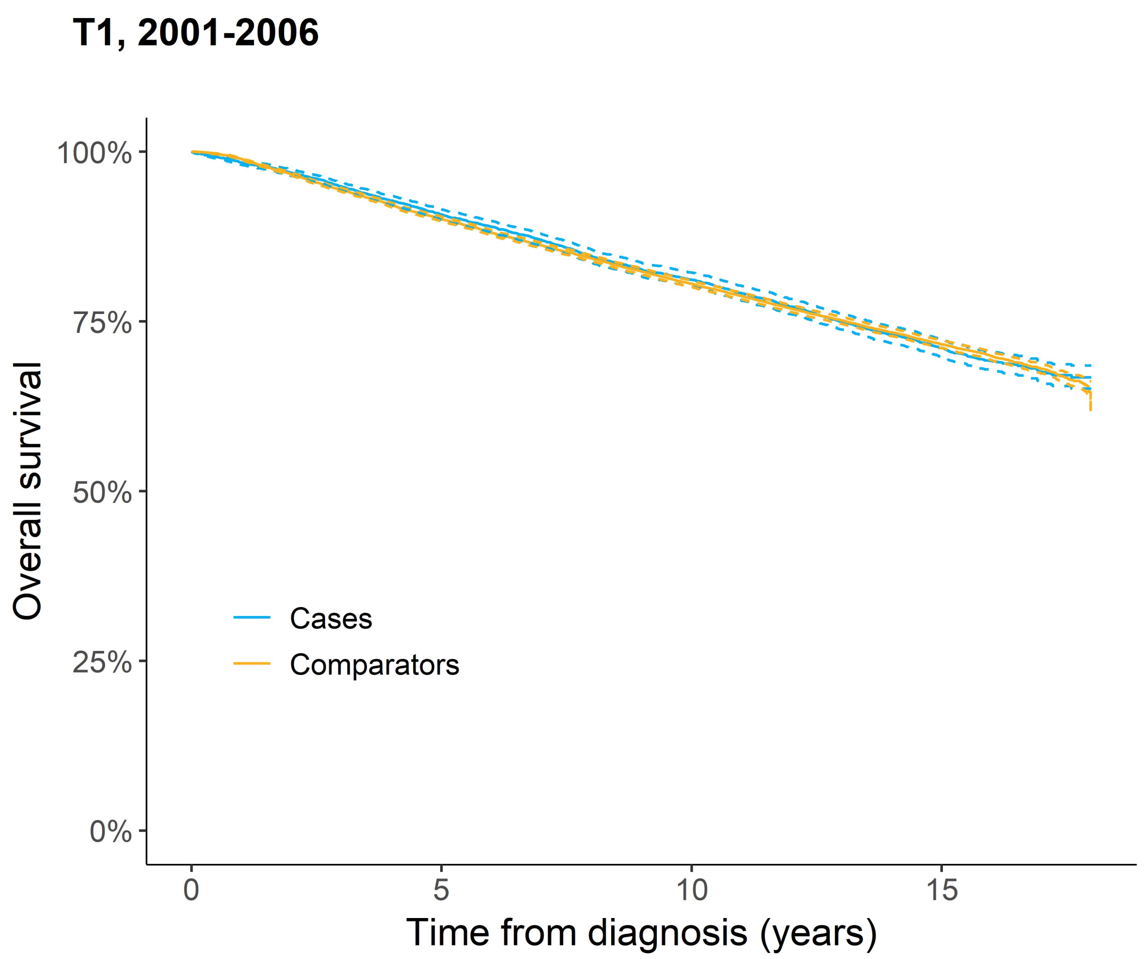

**Cases**	5407	4907	4387	1853
**Comparators**	26596	23961	21427	9216

Patients at risk

Supplementary Figure 2. Overall survival: Patients with thin (≤ 1.0 mm) cutaneous malignant melanoma as first diagnosis of malignant melanoma reported in the Malignant Melanoma Database Sweden (MMBaSe) between 2001 and 2006 (blue) and their matched comparators (yellow). Dashed lines represent 95% CI.

T1, 2007-2012

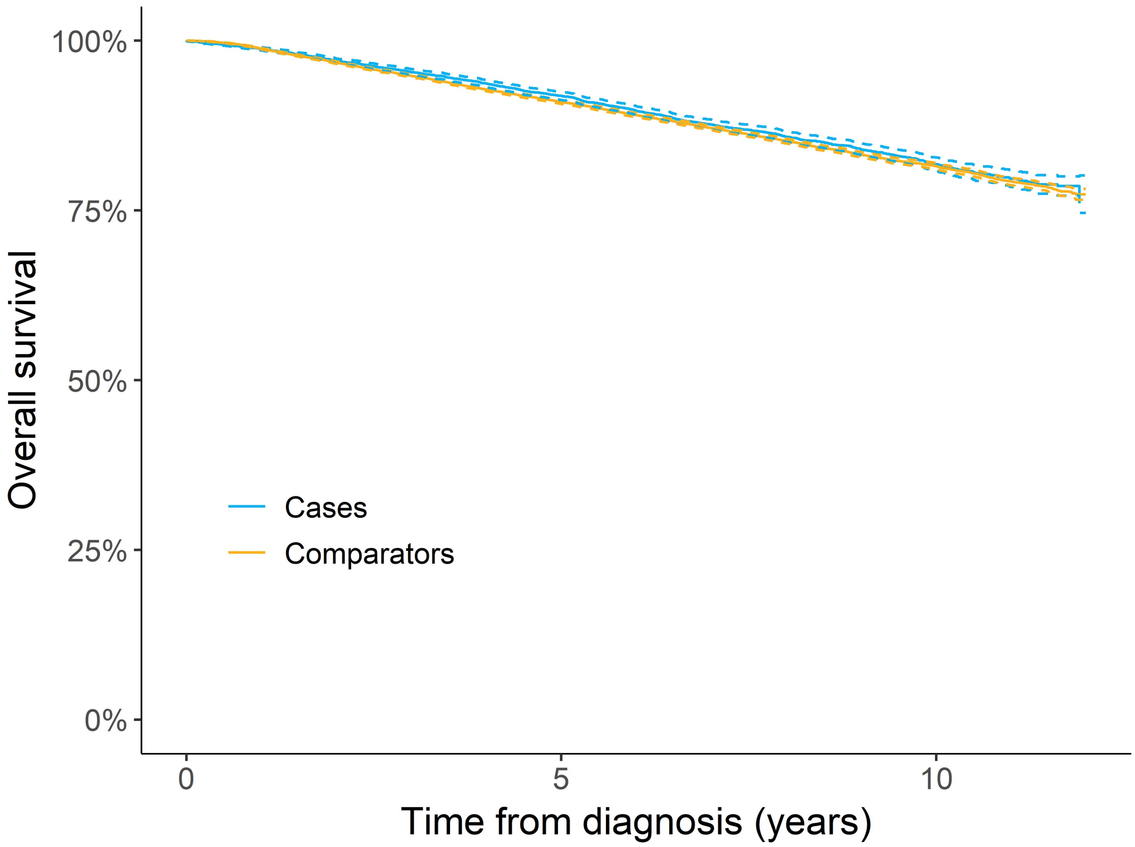

**Cases**	8097	7440	1847
**Comparators**	39907	36306	9038

Patients at risk

Supplementary Figure 3. Overall survival: Patients with thin (≤ 1.0 mm) cutaneous malignant melanoma as first diagnosis of malignant melanoma reported in the Malignant Melanoma Database Sweden (MMBaSe) between 2007 and 2012 (blue) and their matched comparators (yellow). Dashed lines represent 95% CI.

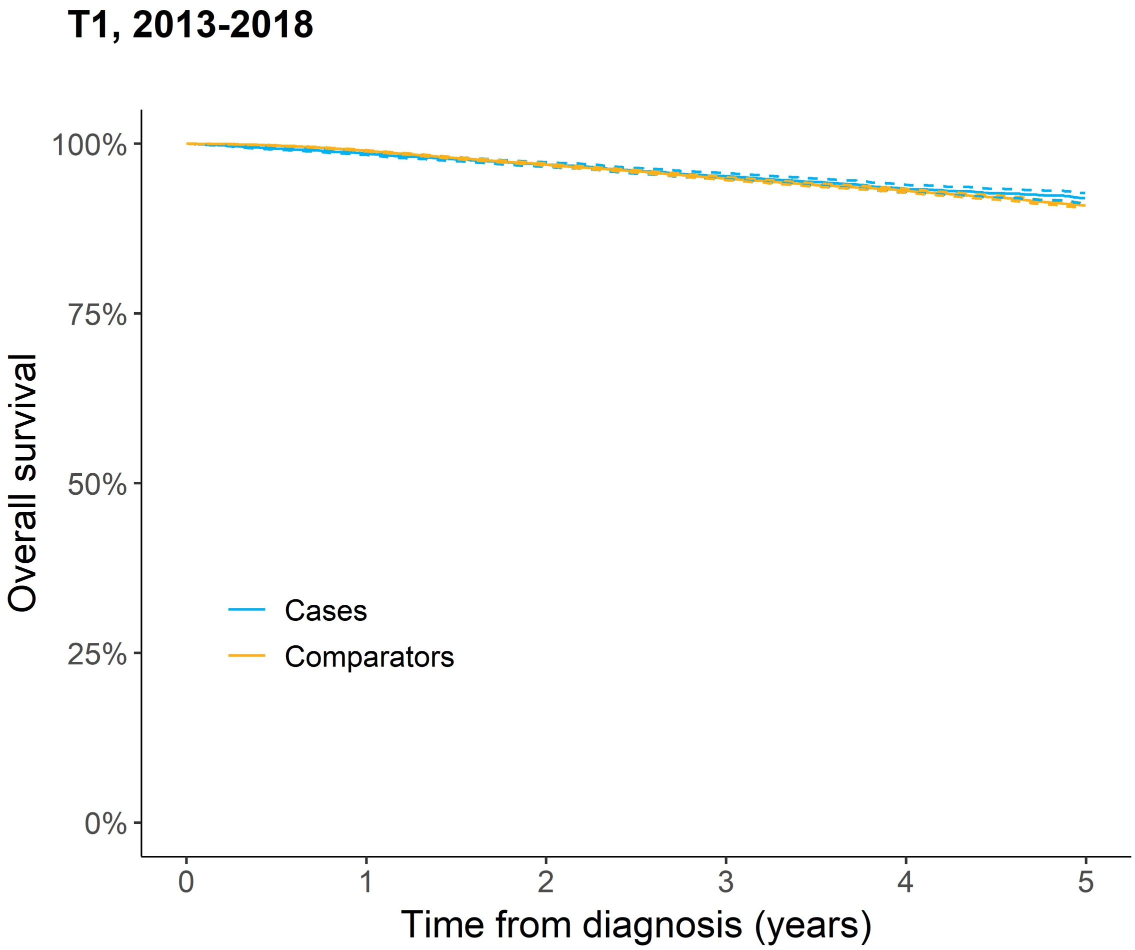

**Cases**	12339	9848	7560	5346	3338	1547
**Comparators**	60880	48735	37214	26254	16377	7543

Patients at risk

Supplementary Figure 4. Overall survival: Patients with thin (≤ 1.0 mm) cutaneous malignant melanoma as first diagnosis of malignant melanoma reported in the Malignant Melanoma Database Sweden (MMBaSe) between 2013 and 2018 (blue) and their matched comparators (yellow). Dashed lines represent 95% CI.

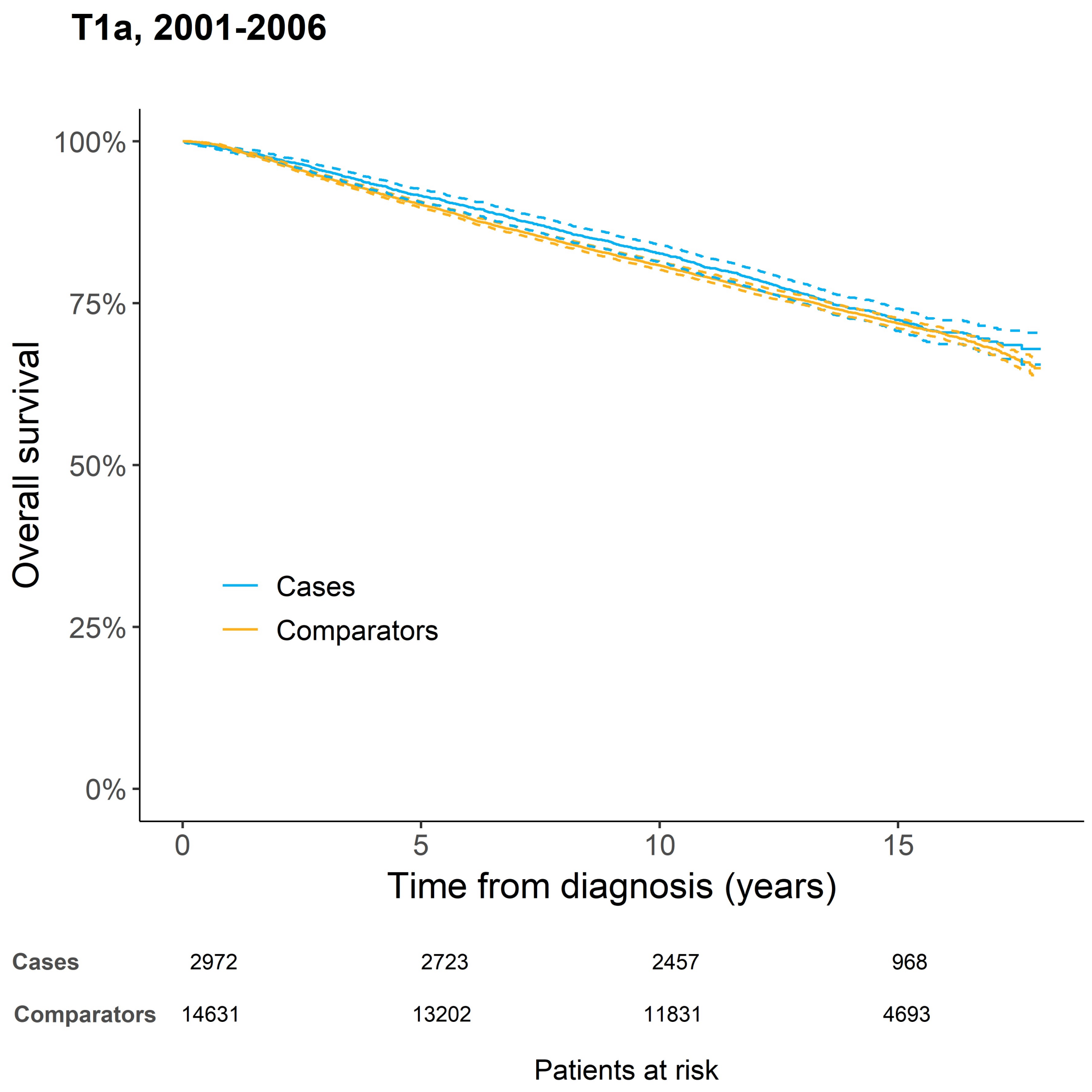

Supplementary Figure 5. Overall survival: Patients with stage T1a as first diagnosis of cutaneous malignant melanoma reported in the Malignant Melanoma Database Sweden (MMBaSe) between 2001 and 2006 (blue) and their matched comparators (yellow). Dashed lines represent 95% CI.

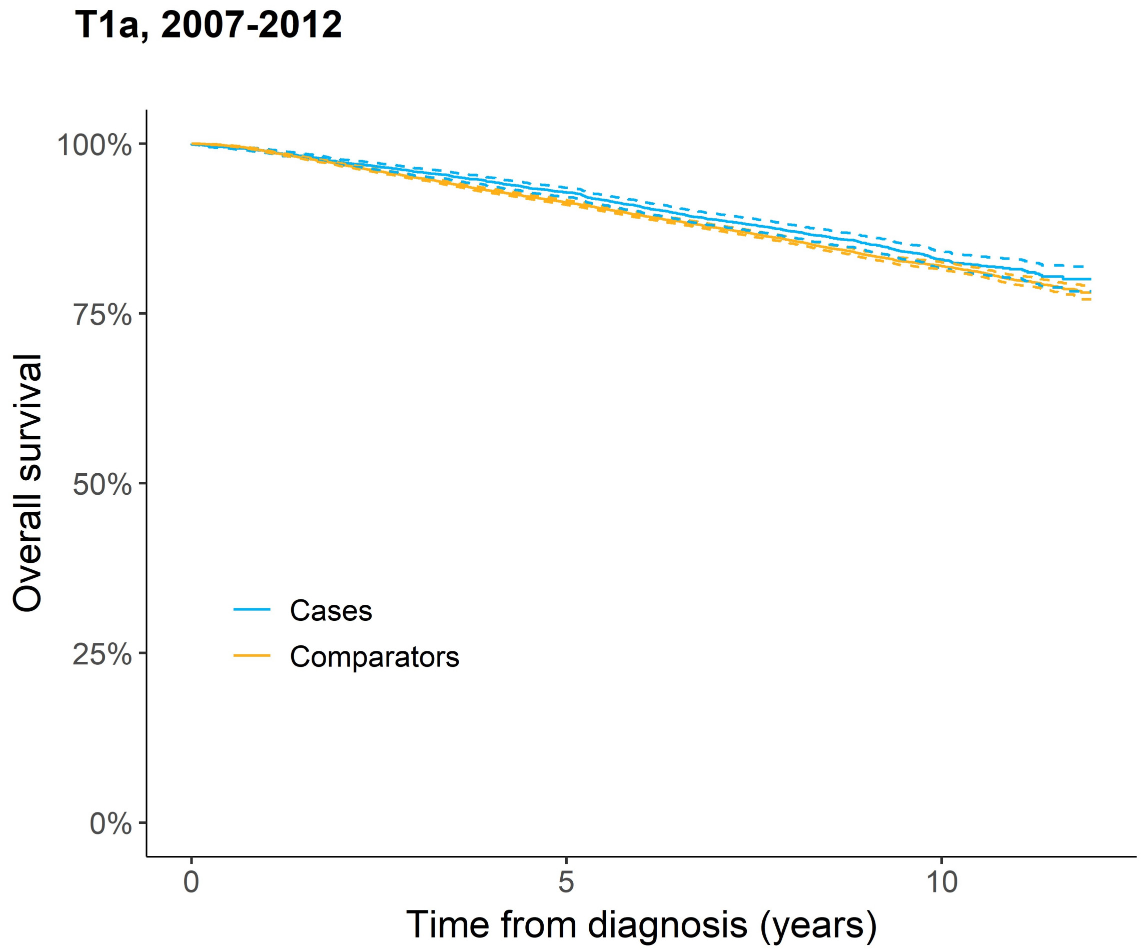

**Cases**	5216	4842	1204
**Comparators**	25705	23484	5838

Patients at risk

Supplementary Figure 6. Overall survival: Patients with stage T1a as first diagnosis of cutaneous malignant melanoma reported in the Malignant Melanoma Database Sweden (MMBaSe) between 2007 and 2012 (blue) and their matched comparators (yellow). Dashed lines represent 95% CI.

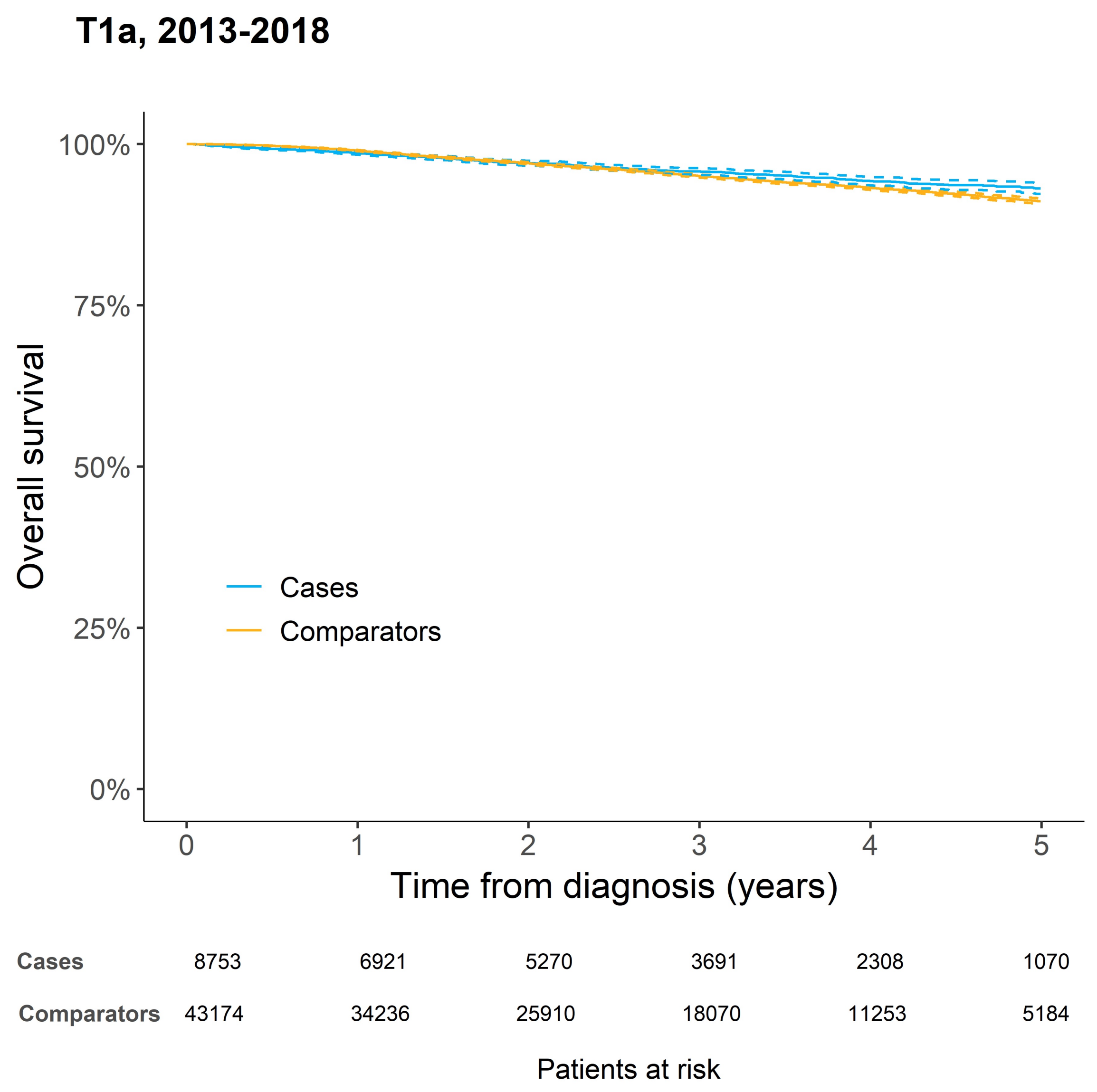

Supplementary Figure 7. Overall survival: Patients with stage T1a as first diagnosis of cutaneous malignant melanoma reported in the Malignant Melanoma Database Sweden (MMBaSe) between 2013 and 2018 (blue) and their matched comparators (yellow). Dashed lines represent 95% CI.
